# Impact of Post-Curing Exposure Time on the Dimensional Fidelity of 3D-Printed Provisional Crowns: A Root Mean Square (RMS) Evaluation

**DOI:** 10.3390/jfb17060263

**Published:** 2026-06-01

**Authors:** Miruna Andreea Anghel, Ioana Mitruț, Mihaela Ionescu, Alexandru Andrei Iliescu, Petre Costin Mărășescu, Cristian Zaharia, Horia Octavian Manolea

**Affiliations:** 1 Department of Dental Prosthesis Technology, Faculty of Dentistry, University of Medicine and Pharmacy of Craiova, 200349 Craiova, Romania; miruna.anghel@umfcv.ro (M.A.A.); petre.marasescu@umfcv.ro (P.C.M.); 2 Department of Medical Informatics and Biostatistics, Faculty of Dentistry, University of Medicine and Pharmacy of Craiova, 200349 Craiova, Romania; mihaela.ionescu@umfcv.ro; 3 Department of Oral Rehabilitation, Faculty of Dentistry, University of Medicine and Pharmacy of Craiova, 200638 Craiova, Romania; alexandru.iliescu@umfcv.ro; 4 Department of Prostheses Technology and Dental Materials, Faculty of Dental Medicine, Victor Babes University of Medicine and Pharmacy, 300041 Timisoara, Romania; cristian.zaharia@umft.ro; 5 Department of Dental Materials, Faculty of Dentistry, University of Medicine and Pharmacy of Craiova, 200349 Craiova, Romania; horia.manolea@umfcv.ro

**Keywords:** 3D printing, post-curing time, RMS deviation, dimensional accuracy, provisional crowns, photopolymerization

## Abstract

Background: Dimensional stability during post-curing exposure time is critical for the clinical success of 3D-printed restorations. This study evaluates how different post-curing protocols affect the accuracy of provisional crowns. Methods: Fifty-four provisional crowns (*n* = 27 incisors; *n* = 27 premolars) were fabricated using an ASIGA 3D MAX UV printer. The crowns were subjected to three post-curing durations (5, 10, and 20 min). Dimensional deviation was quantified using RMS values. Results: RMS values showed a numerical, but not statistically significant, increase with longer post-curing times (*p* > 0.05). The 5 min protocol yielded the lowest descriptive deviations for both tooth types. Conclusions: Although no statistically significant differences were observed, shorter post-curing times were associated with lower RMS values and may help preserve dimensional accuracy. Further studies with larger subgroup sizes are needed to confirm these trends.

## 1. Introduction

Three-dimensional (3D) printing has become an integral component of contemporary digital dentistry, offering rapid fabrication, workflow efficiency, and high reproducibility for provisional and definitive restorations [1,2,3,4]. Among the clinical requirements for provisional crowns, dimensional accuracy remains essential, as deviations from the intended geometry can compromise marginal adaptation, occlusal stability, and long-term performance [5,6,7].

The precision of 3D-printed restorations is influenced by multiple factors, including printer technology, resin composition, build orientation, layer thickness, and post-processing parameters [8,9,10,11]. Post-curing is particularly critical because it enhances the degree of conversion and mechanical properties of photopolymerizable resins. However, extended exposure may also intensify polymerization shrinkage and internal stress development, potentially leading to geometric deformation, especially in thin or unsupported structures [12,13,14].

Previous studies investigating post-curing protocols have reported heterogeneous findings. Some authors describe increased cross-linking and improved mechanical strength with prolonged exposure, accompanied by additional volumetric shrinkage and measurable distortion [12,13,14]. Others report minimal or clinically acceptable dimensional changes across different curing durations, suggesting that the effect of post-curing time may depend on resin chemistry, curing unit characteristics, and total energy dose. For provisional crowns specifically, available evidence remains limited and inconsistent, with many studies evaluating only a single curing protocol or comparing different devices without isolating exposure time as an independent variable [13,15,16,17]. Despite the widespread adoption of 3D printing in restorative dentistry, there is no consensus regarding the optimal post-curing duration required to maintain dimensional fidelity in provisional crowns. The lack of standardized protocols and the conflicting results reported in the literature highlight the need for controlled investigations focusing specifically on the relationship between post-curing exposure time and geometric accuracy.

Therefore, the objective of this study was to evaluate how different post-curing exposure times influence the dimensional accuracy of 3D-printed provisional crowns, quantified through Root Mean Square (RMS) deviation between the printed restorations and their reference CAD designs.

## 2. Materials and Methods

### 2.1. Study Design and Sample Preparation

A total of 54 provisional crowns were fabricated for this experimental study. All crowns were made starting from the same digital design made after scanning an original in-house made didactic model for the Prosthesis Technology laboratories, with standard preparations made for metal ceramic crowns with a chamfer finish line. Two distinct tooth morphologies were investigated—namely, the maxillary central incisor and the maxillary premolar—with 27 crowns assigned to each group. The required sample size was calculated using G*Power 3.1.9.7 (Heinrich Heine University Düsseldorf, Düsseldorf, Germany), for the F-test family, assuming α = 0.05, power = 0.80, and an effect size of 0.5, confirming that a minimum of 52 samples was adequate.

All crowns were designed in Exocad DentalCAD (version 3.1, Rijeka, Exocad GmbH, Darmstadt, Germany) using the “copy tooth mirror” function to ensure standardized morphology, with a cement gap of 0.08 mm at a 1 mm distance from the margin and the following crown border parameters: Horizontal 0.2 mm, Angled 0.3 mm, and Angle 60°.

The STL files were exported for fabrication to the 3D printer.

The virtual crowns were printed using an ASIGA MAX UV printer (Asiga, Sydney, Australia) with ACCUPRINT C&B resin (D-Tech Dental Technologies, Pune, India). All provisional crowns were oriented at 45° with the cervical margin facing upward and manufactured under controlled laboratory conditions (23 ± 1 °C; 50 ± 5% humidity).

### 2.2. Cleaning and Post-Curing Protocols

After printing, all crowns were cleaned using the MTC Cleaner system (Meccatronicore, Trento, Italy) in isopropyl alcohol (IPA) for 1 min at three standardized intensities: low, medium, and high.

Post-curing was performed in the BBC Compact unit (Meccatronicore, Pergine Valsugana, Italy), which emits narrow-band 405 nm LED light at 12–15 mW/cm^2^. Three preset exposure times were applied:

- 5 min (Lot A)
- 10 min (Lot B)
- 20 min (Lot C)

These correspond to approximate cumulative energy doses of 3.6–4.5 J/cm^2^, 7.2–9.0 J/cm^2^, and 14.4–18.0 J/cm^2^, respectively.

Each combination of tooth type, cleaning intensity, and curing duration resulted in *n* = 3 crowns per subgroup.

### 2.3. Optical Scanning

All printed crowns were scanned using a Medit i600 intraoral scanner (Medit Corp., Seoul, Republic of Korea). Scanning was performed under controlled ambient lighting (<500 lux), with the crowns positioned on a matte surface to minimize reflection. The scanner operated at a point accuracy of 0.02 mm, and calibration was performed daily according to the manufacturer’s instructions.

A single trained operator performed all scans to minimize variability. The resulting STL files were exported to the 3D printer for dimensional analysis.

### 2.4. Dimensional Analysis and RMS Calculation

Dimensional accuracy was evaluated by superimposing each scanned crown onto its corresponding CAD reference model. Alignment was performed in Medit Design software (version 1.3.2) using a standardized two-step registration protocol:

1. Manual pre-alignment using three anatomical landmarks (occlusal, buccal, cervical).
2. Automated global registration using the Iterative Closest Point (ICP) algorithm, which minimizes point-to-surface distances through iterative refinement.

No manual adjustments were applied after ICP alignment. RMS deviation values were automatically calculated as the square root of the mean squared distance between corresponding points of the two datasets.

**Figure 1:**
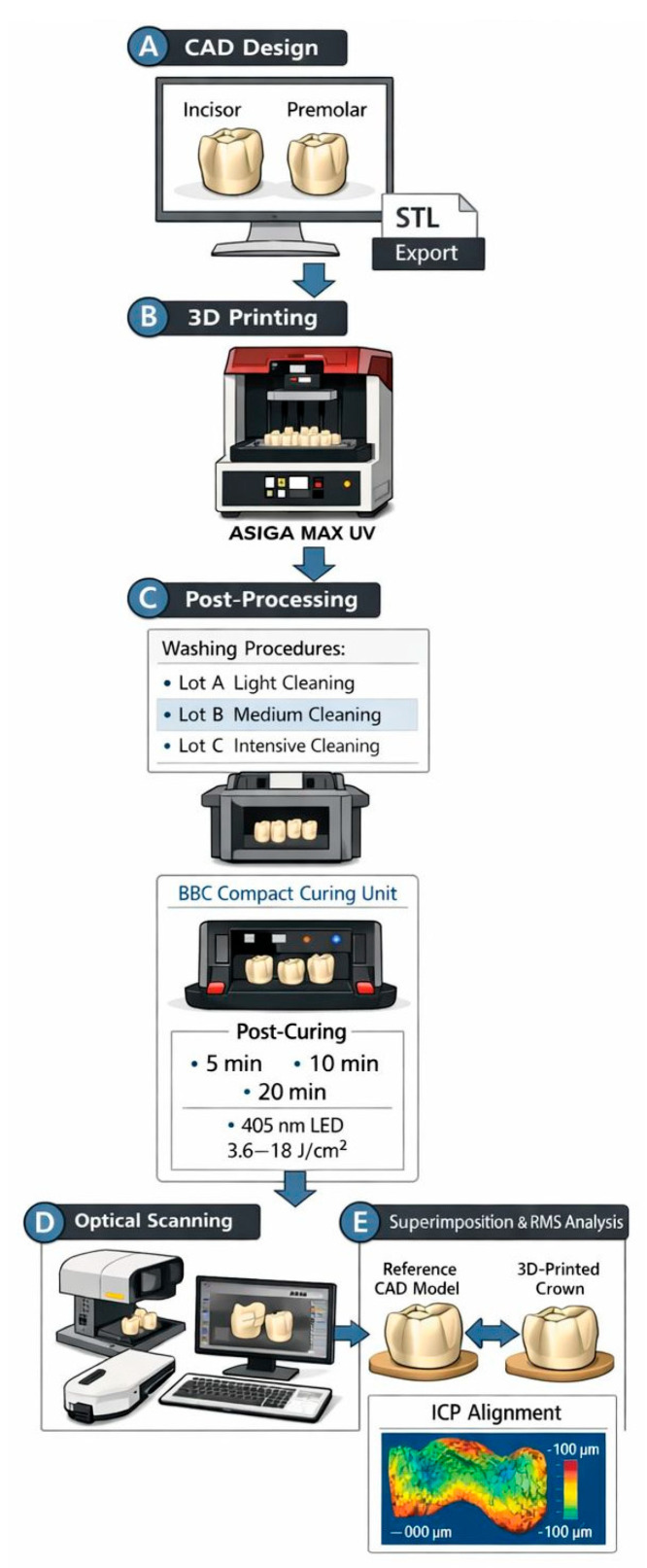
Consolidated workflow of the study. (**A**) CAD design of provisional crowns (incisor and premolar) generated from the reference digital model and exported as STL files. (**B**) 3D printing of all crowns using the ASIGA MAX UV printer with standardized parameters (100 µm layer thickness, biocompatible resin). (**C**) Post-processing protocol, including three cleaning intensities (Lot A: light, Lot B: medium, Lot C: intensive) followed by post-curing for 5, 10, or 20 min in the BBC Compact curing unit (405 nm LED; 3.6–18 J/cm^2^). (**D**) Optical scanning of printed crowns using the Medit i700 scanner to obtain STL datasets for dimensional evaluation. (**E**) Superimposition of printed crowns onto the reference CAD model using manual point selection, followed by ICP global registration, and calculation of RMS deviation values with color-mapped error visualization.

To verify measurement reliability, 6 of the samples were randomly selected to be re-evaluated after two weeks by the same operator. The intraclass correlation coefficient (ICC) was 0.94, indicating excellent intra-observer consistency (Figure 1).

### 2.5. Statistical Analysis

All RMS values were initially processed in Microsoft Excel (Microsoft Corporation, Redmond, WA, USA) to compute descriptive statistics (mean and standard deviation). Statistical analysis was performed using SPSS version 26 (IBM Corp., Armonk, NY, USA).

Normality was assessed using the Shapiro–Wilk test, and homogeneity of variances using Levene’s test.

Depending on the comparison, the following statistical tests were used:

- One-way ANOVA was used to evaluate differences within each curing duration across cleaning intensities.
- Two-way ANOVA assessed the effects of curing time, cleaning intensity, and their interaction.
- Tukey’s HSD test was planned for post hoc comparisons when applicable.

The significance threshold was set at α = 0.05.

## 3. Results

A total of 54 provisional crowns were analyzed. RMS deviation values were used to quantify dimensional accuracy by comparing each printed crown with its corresponding CAD reference model. Across all experimental conditions, RMS values increased numerically with longer post-curing times; however, none of these differences reached statistical significance.

### 3.1. Incisors

For incisors, RMS values showed a progressive numerical increase from 5 to 20 min of post-curing (Table 1).

Cleaning intensity (low, medium, high) produced only minor intra-group variations.

One-way ANOVA revealed no statistically significant differences within any curing duration:

- Lot A (5 min): *p* = 0.729
- Lot B (10 min): *p* = 0.988
- Lot C (20 min): *p* = 0.709

**Figure 2:**
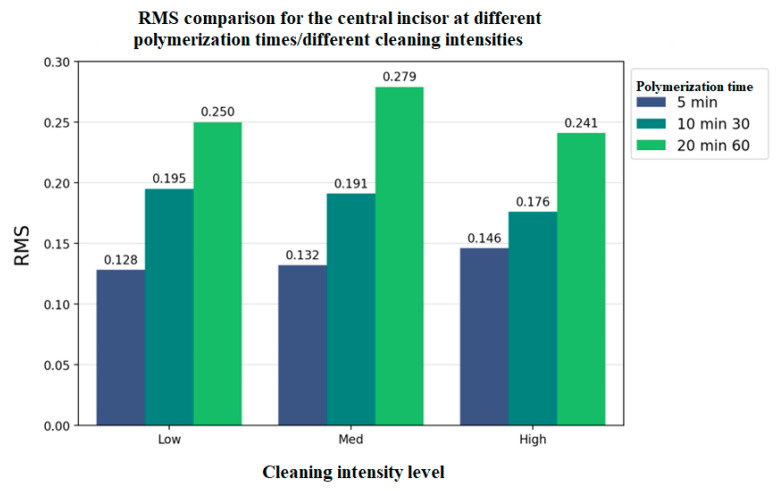
Central incisor RMS comparison for the 3 lots.

Cleaning intensity produced minor intra-group variations without altering the overall time-dependent trend. At each intensity level investigated (respectively, low, medium and high), an obvious progression of the magnitude represented by the visual height of the graphic elements is observed, directly proportional to the increase in exposure time. This observation indicates a direct and consistent relationship between duration and the measured response, regardless of the intensity level (Figure 2).

This probably indicates significant shrinkage of the material, resulting in a greater deviation from the design. Within the limits of the test, prolonging the curing time and increasing the intensity increases the value (RMS) of the deviation from the design of the central incisor.

For all three lots (A, B and C), a one-way ANOVA was conducted to determine if the measured RMS was different for the crowns that were light cured at different preset programs with three types of cleaning (low, medium, and high). There were no outliers, as assessed by boxplot; data was normally distributed for each group, as assessed by the Shapiro–Wilk test (*p* > 0.05), and there was homogeneity of variances, as assessed by Levene’s test of homogeneity of variances (*p* > 0.05). RMS measurements for incisors are presented as mean ± SD in Table 1.

For Lot A, the mean RMS measurements increased from the low group to the medium group and to the high group, in this order; however, the differences between groups were not statistically significant, *p* = 0.729 (Table 1). For Lot B, the mean RMS measurements decreased from the low group to the medium group and to the high group, in this order; however, the differences between groups were not statistically significant, *p* = 0.988 (Table 1). For Lot C, the mean RMS measurements increased from the low group to the medium group, but then decreased from the medium group to the high group; the differences between groups were not statistically significant, *p* = 0.709 (Table 1).

Similar tests between the three lots, for each cleaning type (low, medium and high) yielded similar results, with no statistically significant differences between lots, *p* > 0.05 (Table 1).

### 3.2. Premolars

For the premolar, short curing times (5 and 10 min) produced comparable RMS values, whereas 20 min resulted in substantially higher deviations.

▪ Lot A (5 min): RMS ranged from 0.080 to 0.114 (mean ≈ 0.097)
▪ Lot B (10 min): RMS values remained below 0.20, with the highest values at medium intensity (mean ≈ 0.187).
▪ Lot C (20 min): RMS ranged from 0.267 to 0.321 (mean ≈ 0.294)

A marked increase was observed between 10 and 20 min, with mean RMS values approximately tripling compared to 5 min curing.

Cleaning intensity had a limited influence compared to curing time.

**Figure 3:**
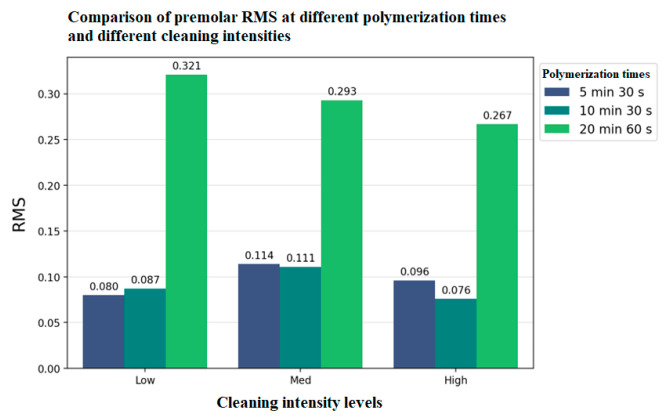
Comparison of premolar RMS at different washing intensities and polymerization durations/times.

From 5 min to 10 min, there is no real change in the deviation. The transition of the values to 20 min of light curing drastically changes the profile; all increase to >0.26, and the average reaches ≈ 0.294, i.e., three times higher than at short times (Figure 3).

For all three lots (A, B and C), a one-way ANOVA was conducted to determine if the measured RMS was different for the crowns that were light cured at different preset programs with three types of cleaning (low, medium, and high). There were no outliers (no Z-scores higher than ±3) and the data was normally distributed for each group, as assessed by the Shapiro–Wilk test (*p* > 0.05). There was homogeneity of variances, as assessed by Levene’s test of homogeneity of variances (*p* > 0.05). RMS measurements for the premolars are presented as mean ± SD (Table 2).

For Lot A, the mean RMS measurements increased from the low group to the medium group, then decreased from the medium group to the high group; however, the differences between groups were not statistically significant, *p* = 0.326 (Table 2). For Lot B, the mean RMS measurements increased from the low group to the medium group, and then decreased to the high group; the differences between groups were not statistically significant, *p* = 0.875 (Table 2). For Lot C, the mean RMS measurements decreased from the low group to the medium group to the high group, in this order; the differences between groups were not statistically significant, *p* = 0.974 (Table 2).

Similar tests between the three lots, for each cleaning type (low, medium and high) yielded similar results, with no statistically significant differences between lots, *p* > 0.05 (Table 2).

### 3.3. Comparison Between Tooth Types

Mean RMS values for incisors and premolars at each curing time are summarized in Table 3 (Table 3).

- At 5 min, premolars showed lower RMS values than incisors.
- At 10 min, incisors exhibited lower RMS values than premolars.
- At 20 min, both tooth types showed the highest RMS values, with premolars slightly higher.

These observations were based on descriptive comparisons. Since no significant differences were identified among the cleaning intensities, the subsequent analysis was focused primarily on the effect of post-curing time.

**Figure 4:**
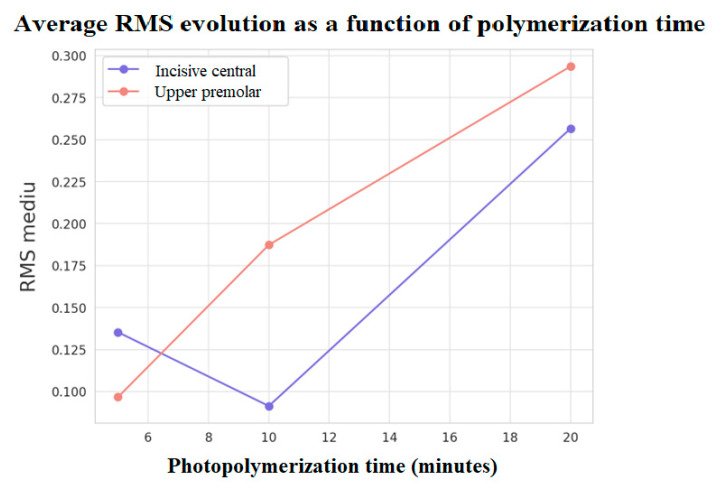
Evolution of RMS as a function of light curing time.

Optimal results in terms of deviation from design, times of 5 min (Lot A) are optimal for both types of teeth; 10 min provides better results for incisors and may be acceptable for premolars, while at 20 min., deviation from design begins to be significant for both types of teeth (Figure 4).

### 3.4. Two-Way ANOVA

A two-way ANOVA was conducted to examine the effects of the curing time (5, 10, 20 min) and the cleaning intensity (low, medium, high) on the RMS values for incisors and premolars. Potential outliers were assessed by the computation of Z-scores; normality was assessed using Shapiro–Wilk’s normality test for each cell of the design, and homogeneity of variances was assessed by Levene’s test. Following this analysis, no outliers were identified, residuals were normally distributed (*p* > 0.05), and homogeneity of variances (*p* = 0.059) was also identified.

For incisors, the interaction effect between the curing time and the cleaning intensity on RMS values was not statistically significant, F(4,18) = 1.049, *p* = 0.064, partial η^2^ = 0.625. Still, an analysis of the main effect for both parameters was performed, which indicated that the main effects were independently not statistically significant, F(2,18) = 2.808, *p* = 0.172, partial η^2^ = 0.544 for the cleaning intensity, and F(2,18) = 1.311, *p* = 0.095, partial η^2^ = 0.869 for the curing time.

The ANOVA analysis did not reveal significant differences in RMS values (for incisors or premolars) depending on the cleaning intensity and curing time. There are no statistically significant interaction effects between them (Table 4).

For premolars, the interaction effect between the curing time and the cleaning intensity on RMS values was not statistically significant, F(4,18) = 1.184, *p* = 0.351, partial η^2^ = 0.208. Still, an analysis of the main effect for both parameters was performed, which indicated that the main effects were independently not statistically significant, F(2,18) = 3.120, *p* = 0.187, partial η^2^ = 0.503 for the cleaning intensity, and F(2,18) = 1.801, *p* = 0.194, partial η^2^ = 0.167 for the curing time.

Thus, although RMS values increased numerically with longer post-curing exposure, these differences were not statistically significant.

## 4. Discussion

This study investigated the influence of different post-curing exposure times on the dimensional accuracy of 3D-printed provisional crowns, quantified through RMS deviation. Although RMS values increased numerically with longer curing durations, no statistically significant differences were detected across any experimental condition. These findings indicate that, within the limits of the present methodology, post-curing time did not produce measurable changes in dimensional fidelity.

The descriptive trend observed—namely, higher RMS values at 20 min compared with 5 and 10 min—is consistent with previous reports suggesting that prolonged post-curing may intensify polymerization shrinkage or internal stress formation in photopolymerizable resins [12,13,14]. However, because the present study did not directly assess volumetric shrinkage or stress development, these mechanisms remain hypothetical and should be interpreted cautiously.

In our study, the selection of 5, 10, and 20 min post-curing durations was based on a combination of manufacturer recommendations and previously published studies evaluating the influence of curing time on the mechanical and dimensional properties of 3D-printed dental resins. The 5 min cycle represents the minimum exposure recommended for achieving adequate polymerization, while the 10 and 20 min cycles reflect extended curing protocols commonly used in clinical and laboratory settings to enhance material strength and surface hardness. These intervals also align with the curing times investigated in earlier research, allowing meaningful comparison with existing literature and ensuring that the selected durations represent clinically relevant post-processing conditions [18].

For both incisors and premolars, the lowest deviations were recorded at 5 min, while the highest occurred at 20 min. Premolars exhibited slightly higher RMS values than incisors at longer curing times. This pattern may reflect morphological differences between tooth types, as larger or more complex geometries may be more susceptible to cumulative polymerization effects. While the subgroup size may be considered as limiting the strength of this observation, the appropriate overall effect size confirms it.

Previous studies have reported heterogeneous outcomes regarding the impact of post-curing duration on dimensional accuracy. Some authors describe increased cross-linking and improved mechanical properties with extended curing, accompanied by additional volumetric shrinkage and geometric distortion [19,20,21]. Other investigations found minimal or clinically acceptable dimensional changes across different curing protocols, suggesting that resin chemistry, curing unit characteristics, and total energy dose may modulate the effect [13,15,16,17].

The present findings align with studies reporting that dimensional deviations may increase with prolonged curing but remain within clinically acceptable limits. The absence of statistically significant differences in this study supports the notion that post-curing time alone may not be a dominant factor influencing accuracy, particularly when standardized printing and cleaning protocols are used.

Another factor that may have contributed to the measured deviations is the superimposition method used to align the printed crowns with the reference CAD model. Although the iterative closest point (ICP) algorithm provides a widely accepted and robust approach for 3D dataset alignment, the procedure still involves an initial manual point selection step, which may introduce small operator-dependent variations. Furthermore, ICP minimizes the overall point-to-surface distance but may propagate local mismatches depending on surface geometry and the distribution of reference points. These methodological characteristics can generate minor alignment-related discrepancies that are subsequently reflected in the RMS values. Therefore, part of the dimensional deviation observed in this study may be attributed not only to post-curing effects but also to intrinsic limitations of the superimposition process.

Dimensional accuracy is essential for the clinical performance of provisional crowns, as deviations may affect marginal fit, occlusal stability, and patient comfort [5,6,7]. Although the numerical increase in RMS values at 20 min suggests a potential reduction in accuracy, the lack of statistical significance indicates that all three curing protocols produced comparable outcomes under controlled laboratory conditions. Clinicians may, therefore, prioritize workflow efficiency without compromising dimensional fidelity, particularly when shorter curing times are compatible with the resin manufacturer’s recommendations.

Another factor that may influence dimensional accuracy is the intrinsic behavior of the photopolymerizable resin used in this study. The degree of conversion, cross-linking density, and polymer network architecture vary among commercially available resins and can affect their susceptibility to post-curing shrinkage. Materials with higher monomer mobility or lower initial conversion may undergo more pronounced volumetric changes during post-curing, potentially amplifying geometric distortion. Additionally, the presence of fillers, photoinitiator concentration, and resin viscosity can influence polymerization kinetics and the extent of post-curing contraction. These material-dependent characteristics may therefore contribute to the dimensional deviations observed and should be considered when comparing results across studies using different resin systems [22].

Future studies incorporating direct temperature monitoring or comparing different thermal profiles would help clarify the extent to which curing temperature contributes to dimensional deviation [23,24]. Precisely controlling post-curing parameters, such as temperature and duration, is critical, as these factors significantly influence the dimensional accuracy of printed dental components [25,26]. The duration of post-polymerization further modulates surface roughness and color stability, both of which can indirectly impact the long-term dimensional integrity of dental restorations [27]. While extended curing times generally improve mechanical properties, such as hardness and fracture toughness, over-curing can negatively impact polymerization efficiency and chemical structure, highlighting the need for precisely calibrated post-curing regimens [28]. The type of 3D printer utilized for fabricating dental appliances can also influence the mechanical properties and dimensional stability of the printed object, while post-curing conditions and atmosphere may further affect material wear behavior and long-term performance, necessitating further investigation into printer-specific post-curing adjustments [29,30].

### 4.1. Limitations

Several limitations must be acknowledged.

First, the small subgroup size (*n* = 3 per condition) may limit statistical power, potentially masking true differences between curing protocols.

Second, the study evaluated only one resin type and one curing device; results may vary with alternative materials or light-curing technologies.

Third, the study focused exclusively on external geometry; internal fit, marginal adaptation, and mechanical properties were not assessed.

Finally, the mechanisms underlying the observed numerical trends—such as polymerization shrinkage or internal stress—were not directly measured.

### 4.2. Future Directions

Future research should incorporate larger sample sizes, multiple resin formulations, and diverse curing units to better characterize the relationship between post-curing parameters and dimensional stability. Complementary analyses, such as volumetric shrinkage measurements, finite element modeling, or micro-CT evaluation of internal fit, may provide deeper insight into the mechanisms driving dimensional changes.

The decision to include three cleaning intensities (low, medium, high) was based on the manufacturer’s recommendations for the tested resin and on prior studies suggesting that solvent exposure, agitation, and cleaning dynamics may influence polymerization behavior and dimensional accuracy in 3D-printed resins [17]. Cleaning protocols have been reported to affect residual monomer removal, surface integrity, and the degree of conversion, potentially interacting with post-curing kinetics [23].

In the present study, however, cleaning intensity did not produce statistically significant differences in RMS values across any curing duration. Although the variable did not demonstrate a measurable effect, its inclusion provides preliminary insight into the robustness of the material’s behavior and highlights the need for future studies with larger sample sizes or alternative cleaning protocols to fully elucidate its potential influence.

## 5. Conclusions

Within the limitations of the present study, post-curing exposure time did not exert a statistically significant influence on the dimensional accuracy of 3D-printed provisional crowns, as evaluated by RMS deviation from the reference CAD designs. These findings suggest that, under the investigated experimental conditions, variations in curing duration alone may not represent a critical determinant of geometric fidelity. Rather, the dimensional accuracy of additively manufactured provisional restorations is likely governed by a multifactorial interplay of material properties, printing parameters, and post-processing conditions.

## Figures and Tables

**Table 1:** RMS measurements for incisors.

Lot	Low	Medium	High	F (2,6)	*p* *
	Mean ± SD	Mean ± SD	Mean ± SD		
Lot A (5 min)	0.128 ± 0.113	0.132 ± 0.232	0.146 ± 0.131	0.332	0.729
Lot B (10 min)	0.195 ± 0.168	0.191 ± 0.161	0.176 ± 0.159	0.014	0.988
Lot C (20 min)	0.250 ± 0.231	0.279 ± 0.371	0.241 ± 0.241	0.363	0.709
F(2.6)	0.355	0.2266	0.211		
*p* *	0.715	0.803	0.815		

* = One-way ANOVA.

**Table 2:** RMS measurements for premolars.

Lot	Low	Medium	High	F (2,6)	*p* *
	Mean ± SD	Mean ± SD	Mean ± SD		
Lot A (5 min)	0.080 ± 0.071	0.114 ± 0.270	0.096 ± 0.086	1.357	0.326
Lot B (10 min)	0.087 ± 0.079	0.111 ± 0.098	0.076 ± 0.073	0.136	0.875
Lot C (20 min)	0.321 ± 0.315	0.293 ± 0.293	0.267 ± 0.254	0.026	0.974
F(2.6)	1.532	0.562	1.284		
*p* *	0.290	0.597	0.343		

* = One-Way ANOVA.

**Table 3:** RMS mean values (±SD) by tooth type and curing time.

Tooth Type	5 min	10 min	20 min
Central Incisor	0.135 ± 0.009	0.091 ± 0.018	0.257 ± 0.020
Premolar	0.097 ± 0.170	0.187 ± 0.010	0.294 ± 0.027

**Table 4:** Results of the ANOVA factorial analysis for the bonding system and the type of bracket used.

Crown	Incisors–RMS			Premolars–RMS		
	F(2,18)/F(4,18)	*p* *	Partial η^2^	F(2,18)/F(4,18)	*p* *	Partial η^2^
Cleaning intensity	2.808	0.172	0.544	3.120	0.187	0.503
Curing time	1.311	0.095	0.869	1.801	0.194	0.167
Interaction effect	1.049	0.064	0.625	1.184	0.351	0.208

* = Two-way ANOVA.

## Data Availability

The original contributions presented in the study are included in the article, further inquiries can be directed to the corresponding author.
